# Nursing students’ perceptions and experiences of e-internships during the COVID-19 pandemic: A phenomenological study

**DOI:** 10.1371/journal.pone.0273963

**Published:** 2022-09-09

**Authors:** Qi-Feng Yi, Jin Yan, Huang Hui, Yan Yang

**Affiliations:** 1 Nursing Department, The Third Xiangya Hospital of Central South University, Changsha, China; 2 XiangYa Nursing School of Central South University, Changsha, China; 3 Department of Cardiovascular, The Third Xiangya Hospital of Central South University, Changsha, China; University of Auckland, NEW ZEALAND

## Abstract

**Background:**

Clinical internship is an indispensable stage for nursing students to graduate successfully and become qualified nurses. However, COVID-19, a novel coronavirus disease with strong human-to-human transmission, hit China in late 2019 and forced the Chinese government to suspend classes and clinical internships. To cope with this situation, e-internship, which facilitate varied interactions without the need for direct contact, is used as an alternative strategy to help nursing students continue their internships.

**Objectives:**

To describe the perceptions and experiences of undergraduate nursing students in e-internships during the COVID-19 pandemic.

**Methods:**

A descriptive phenomenological design was adopted. Seventeen undergraduate nursing students in a major teaching hospital in Changsha, China, were recruited into the study. Data were collected through semi-structured, in-depth, face-to-face interviews. The interviews were transcribed verbatim and analyzed using Colaizzi’s approach.

**Results:**

Four themes were captured from the data analysis: perceived images of clinical nurses in e-internships, psychological experience, perceived benefits of e-internships, and perceived limitations of e-internships.

**Conclusions:**

Our findings suggest that e-internship is a suitable method for training and cultivating undergraduate nursing students during a crisis. To enhance the efficiency of e-internships, guidelines and standards should be formulated, and effective measures should be taken to build better e-internship platforms. In the future, we suggest combine on-site internships with e-internships, thereby fully using their advantages, and improve the efficiency of internships as much as possible.

## Introduction

In China, nursing education comprises three main stages: fundamental knowledge stage, professional knowledge stage, and clinical internship stage [1, 2]. The clinical internship is an indispensable part of nursing education. It aims to teach students the necessary knowledge and skills to prepare them for the demanding experience of a registered nurse and gain competence in future work [3]. During internships, fourth-year nursing students will be assigned to different departments to attend face-to-face interactive tutorials conducted by different clinical nursing teachers. Only after completing a clinical internship lasting for at least 10 months can these students graduate and take the licensure exam to become registered nurses [4]. In China, nursing students generally start their clinical internships in April or May. However, in year 2020, the COVID-19 pandemic prevented them from participating in any clinical activity, making it impossible for them to complete school as usual [5].

Electronic learning (e-learning) is an educational system that provides educational or training programs for students or trainees anytime and anywhere by using interactive information and communication technology, such as the internet, TV channels, e-mail, computers, and teleconferencing, synchronously or asynchronously [6]. With its accessibility, flexibility and convenience in facilitating varied interactions without the need for direct contact, e-learning has been used as an alternative strategy to help students continue their learning [7–10].

Early in the epidemic, 103 nursing students in our hospital were in the awkward position of not being able to advance their clinical practice. Fortunately, the hospital nursing teaching and research office then quickly issued the electronic nursing internship program and the online internship syllabus, which were all based on e-learning strategies [11]. Teaching content, methods, schedule, assessment methods, and so on, were made clear. The office suggested that: (1) lectures be delivered online by using the video conferencing platform Tencent Meeting or Dingtalk. On these platforms, virtual rooms can be created, and 300 people can be accommodated at the same time to share the computer screen and various computer files (e.g., documents, PDFs, PowerPoint files), exactly as if the files were projected by video in a lecture hall. (2) clinical nursing skills demonstration be replaced by online demonstration or videos. (3) inquiry of information, providing health education, etc., be carried out through live broadcast. Accordingly, each department made a specific e-internship arrangement based on each department’s characteristics. From July to August 2020, 103 nursing students all participated in e-internships and completed them.

This was the first time in China that conventional education activities had been suspended for so long and nursing students had to depend on e-internships to complete their studies. Therefore, e-internship was a special experience for these students. Although some research had been conducted on nursing education during a pandemic [7, 10, 12], a body of knowledge on internship experiences, especially on e-internship experiences during a pandemic, is lacking. The purpose of this study was to explore the experiences and feelings of undergraduate nursing students carrying out an eight-week e-internships during the COVID-19 pandemic.

## Methods

### Design

Descriptive phenomenology was selected for the current study. The focus of phenomenological research is to describe commonalities of experiences across a population [13]. This method offers us the methodological tools to refrain from our judgments and focus on describing the experience as opposed to interpreting it [14].

### Setting and participants

The study was conducted in a large teaching hospital in Changsha, China. The inclusion criteria for participation in the study were as follows: (1) being a fourth-year undergraduate nursing student, (2) having completed an eight-week e-internship from July to August 2020 in the hospital, and (3) being willing and able to articulate his or her experience and feelings. Participants were recruited using a combination of convenience, snowball and purposive sampling strategies. Our first five participants responded to a recruiting poster distributed to the intern WeChat groups by the head of the clinical teachers. The rest of the participants were recruited through snowball sampling. To maximize the sample diversities and generate rich perspectives, participants were recruited from as many different departments as possible. In agreement with Braun and Clarke’s concept about “saturation”, we believe that data saturation is conditional and there are always new theoretical insights to be made as long as data continues to be collected and analysed. Therefore, when to stop data collection is subjective and depends on the restrictions that we set, for example, time, expense and even our expectations and so on, and cannot be determined(wholly) by in advance of analysis [15]. Therefore, in this study, instead of using traditional data saturation to determine the sample size, we set a rough range (8–16 interviews) based on recommendations from Namey et al. [16].

### Ethics approval and consent to participate

All participants were informed about the details of the study. Participation in the study was voluntary, and participants could withdraw from the study at any time without any consequences. Written consent was obtained from all participants before the initial interview. The anonymity of participants was guaranteed. To ensure confidentiality, participants were provided with an encrypted code. Only researchers had access to research data. The study was conducted following the Declaration of Helsinki.

Our study was part of the research project "The establishment and empirical study of nursing staff’s ability to deal with infectious disease emergencies", and the protocol described in the study was specifically approved by the institutional review board of the Third Xiang-ya Hospital of Central South University. (No: Quick I20011).

### Data collection and data analysis

**Fig 1:**
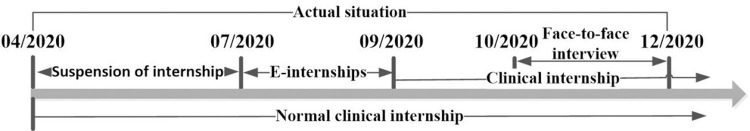
Flow diagram of the study.

In this study, data collection and analysis were performed simultaneously during the study period. Semi-structured, in-depth, face-to-face interviews were conducted from October to December 2020 (Fig 1). To minimize the biases of researchers and participants and to maintain neutrality, a master student in nursing who had no direct relationship with the study but had experience in qualitative interviews was recruited to conduct all interviews, and any suggestions from interviewers were avoided. All interviews were conducted in a private, quiet conference room to maintain privacy and comfort. The interview guide was formulated based on the purpose of the study. Before the study, the interview guide was pilot tested in three preliminary interviews to ensure all questions were clear and understandable to the participants. The final interview outline is shown in Table 1. Before the interview, demographic information, such as age, gender, place of residence, and internship status, was acquired. Each interview lasted 30 to 40 minutes and was audio recorded. The audio recording was transcribed verbatim by two members of the research team within 24 hours, and the interviewer’s reflexive notes were also discussed among members of the research team. The interviews and original transcripts were originally recorded in Chinese. All quotations were translated into English by Guoli Yang (a nursing PhD with six months of study experience in the United States) and back translated into Chinese by Aidi Zhang (a nursing PhD with one year of study experience in the United States) to assess semantic equivalence between the original and back-translated versions of the transcripts.

**Table 1:** Interview guide.

1 Can you tell me your E-internship experiences?
2 How did you learn during your E-internship? Can you share what you have learned and your feelings with me?
3 How did your E-internship, compared to a traditional internship, affect you academically, socially, and psychologically?
4 What challenges did you experience during your E-internship? In what areas do you think e-internships need to be improved?

All audio and written data were imported into the qualitative software NVivo V.12 (QRS International). Based on interview transcripts and field notes, data were analyzed using Colaizzi’s (1978) analytic method [17], which comprises a seven-step, intensive and inductive process: 1) reading the contents of the transcribed interviews repeatedly to identify the overall themes; 2) extracting significant statements while marking potentially essential content; 3) formulating the meanings of significant statements; 4) categorizing the formulated meanings into sub-themes, themes, and clusters of themes; 5) integrating the findings into an exhaustive description by using continuous analysis and a refining process; 6) identifying the fundamental structure through an exhaustive analysis (the fundamental structure was found to consist of 4 dimensions); and 7) verifying the research findings with participants. Initial data coding was completed by two members of the research team. Nevertheless, there were ongoing discussions among all research team members about the analysis of the data, and the research team reached an agreement on the final findings presented in this paper. Throughout the paper, we followed the Consolidated Criteria for Reporting Qualitative Research [18].

### Trustworthiness

Several strategies were used to ensure trustworthiness [19]. Credibility was achieved by in-depth interviews followed by peer debriefing. To ensure the dependability of the analysis, the transcripts were independently analyzed by two researchers by bracketing data on preconceived ideas. To ensure transferability, we provided rich and thorough descriptions of participants’ perceptions and experiences by citing the participants’ verbatim statements. Confirmability was established by sharing the essential categories and themes with each participant. Finally, the verbatim transcripts were used to prove the authenticity of the study.

## Results

### Participants

Seventeen participants, including five males and twelve females, were recruited into the study. They came from six provinces, including Hunan, Chongqing, Yunnan, Zhejiang, Anhui, and Jilin. The characteristics of the participants are shown in Table 2.

**Table 2:** Participant characteristics (n = 17).

Participant No.	Gender	Age	Suburban/Urban	Study instruments	Main Study Platform
Participant 1	Female	21	Urban	Computer	Tencent Meeting + Dingtalk
Participant 2	Female	22	Urban	Computer	Tencent Meeting
Participant 3	Female	22	Suburban	Mobile Phone	Tencent Meeting + Dingtalk
Participant 4	Male	21	Suburban	Mobile Phone	Tencent Meeting + Dingtalk
Participant 5	Female	22	Suburban	Computer	Tencent Meeting
Participant 6	Female	20	Urban	Mobile Phone	Tencent Meeting
Participant 7	Male	22	Suburban	Mobile Phone	Tencent Meeting + Dingtalk
Participant 8	Female	19	Suburban	Computer	Tencent Meeting + Dingtalk
Participant 9	Male	21	Urban	Mobile Phone	Tencent Meeting + Dingtalk
Participant 10	Female	22	Urban	Computer	Tencent Meeting +Dingtalk
Participant 11	Female	22	Urban	Computer	Tencent Meeting
Participant 12	Male	21	Suburban	Mobile Phone	Tencent Meeting
Participant 13	Female	21	Suburban	Computer	Tencent Meeting +Dingtalk
Participant 14	Female	22	Suburban	Mobile Phone	Tencent Meeting +Dingtalk
Participant 15	Male	21	Urban	Computer	Tencent Meeting
Participant 16	Female	22	Suburban	Mobile Phone	Tencent Meeting +Dingtalk
Participant 17	Female	19	Urban	Mobile Phone	Tencent Meeting

We captured four generic categories and 12 subcategories about the participants’ perceptions of and experiences in the e-internship (Table 3).

**Table 3:** Main themes and sub-themes identified in the study.

Main themes	Sub-themes
1. Perceived images of clinical nurses in e-internships	1.1 Dedicated and hardworking
	1.2 Professional and skillful
2. Psychological experience	2.1 Regret and anxiety
	2.2 Acceptance and gratitude
	2.3 Uncertainty
3. Perceived benefits of e-internships	3.1 Integration of theory and clinical practice
	3.2 Relatively realistic clinical experience
4. Perceived limitations of e-internships	4.1 Limitations of practice opportunities
	4.2 Deficits in e-teaching ability
	4.3 Deficits in e-teaching resources
	4.4 Limitations of the learning platforms

### Main theme 1: Perceived images of clinical nurses in e-internships

Although these nursing students did not work and learn with clinical nursing teachers in a real clinical environment, they formed a certain understanding of clinical nurses’ images through e-internships.

#### Sub-theme 1: Dedicated and hardworking

This pandemic is not only a challenge for learning of students, but also for teaching of clinical nurses. In order to do this job well, nursing teachers did a lot of preparation work and sacrificed a lot of rest time. All the participants believed that the teachers were dedicated and hardworking.

*“They were very patient and answered our questions timely*, *even when it wasn’t class time” (Participant 1)*.

*“Not only did they have to complete clinical tasks*, *but they also had to complete teaching tasks. All of them worked very hard and sacrificed much break time” (Participant 3)*.

#### Sub-theme 2: Professional and skillful

Obviously, face-to-face communication was blocked in e-internships, putting forward higher requirements for teachers’ ability to transfer knowledge and skills. Fortunately, students spoke highly of clinical nurses’ professional knowledge and skills, not just clinical knowledge and skills, but also teaching skills, communication skills and so on. Many students said that they never knew that clinical nursing teachers are so versatile before e-internships.

*“They are very knowledgeable and able to answer all our questions*. *Moreover*, *the PPT they made is so beautiful*, *and the operation video they shot is so professional*.*”* (Participant 17).

*“It can be seen that patients were willing to accept themselves as teaching models even in e-internships*. *I think this is inseparable from teacher’s meticulous care for patients and good communication skills*. *In my heart*, *they are omnipotent"* (Participant 10).

*“I like Teacher X’s classes the most*. *Her class can bring us into clinical situations and is very attractive"* (Participant 12).

### Main theme 2: Psychological experience

Faced with the sudden epidemic, forced termination of on-site internships and the implementation of unfamiliar e-internships, nursing students indicated a series of psychological changes.

#### Sub-theme 1: Regret and anxiety

Concerning the psychological changes about the cancellation of clinical internships, regret and anxiety are what the nursing students mentioned the most.

*“I felt regretful for not being able to intern as usual*. *I think a clinical internship is the best way to exercise clinical thinking and practical skills”* (Participant 6).

*“Anxious and pitiful*, *I hoped that the epidemic can be controlled and normal internships can be resumed as soon as possible”* (Participant 10).

#### Sub-theme 2: Acceptance and gratitude

Under the circumstances, no e-internship meant no internship. Each interviewee believed that the e-internship was the most suitable arrangement at that time and they thus accepted it and owed a debt of gratitude to the hospital and the teachers.

*“It is the same across the country (no one can go to the hospital for internships); Although slightly regrettable*, *it is acceptable*. *I knew*,*clinical teachers had done their best to provide us with learning opportunities*. *I thank them so much”* (Participant 14).

*“I am full of gratitude and admiration for the teachers*. *They could have avoided internship but they worked tirelessly to prepare classes and give lectures for us*.*”* (Participant 16).

#### Sub-theme 3: Uncertainty

Uncertainty is a common reaction when faced with unexpected and unfamiliar events. Clinical training provides students with security in the learning of nursing care in health care services. Lack of practical operations, direct guidance and supervision, and face-to-face communications with teachers and patients left participants unsure whether they had mastered what they should master, and they were also uncertain about their future employment.

*“Even though I had completed 2 months of e-internships*, *I was not sure about my clinical practice ability*. *I was also concerned about my license registration and employment”* (Participant 5).

*“I watched the video of indwelling needle in veins many times*, *but I had not truly practiced it on patients*. *I didn’t know whether I can do it”* (Participant 6).

Furthermore, the epidemic also increased their sense of uncertainty about their future.

*“I don’t know when the epidemic will end and when normal studies*, *work and life can be restored*. *All these uncertainties make me feel that I have lost control of my life and can only wait passively”* (Participant 13).

### Main theme 3: Perceived benefits of e-internships

Internship is a central element of nursing education, recognized as one of the most important factors for the development of skills. Although these students did not enter the clinic, the participants stated that they benefited greatly from the e-internships.

#### Sub-theme 1: Integration of theory and clinical practice

Nursing students who have almost no clinical experience are eager to know how to use the knowledge learned in school in clinical situations. The students affirmed the teacher’s practice of combining theory with clinical practice.

*“The teachers sorted out common specialty knowledge for us*, *consolidated our theoretical knowledge*, *and often combined the clinical cases to deepen our impression"* (Participant 1).

*“In the Department of Cardiology*, *the teachers helped us review the basic knowledge of electrocardiograms and used the patient’s clinical electrocardiogram to let us recognize it*.*”* (Participant 5).

#### Sub-theme 2: Relatively realistic clinical experience

Being immersed in a real clinical context is extremely important for training and developing nursing students. Students said that nursing teachers had adopted various methods to create a real clinical environment for them as much as possible.

*“The teacher showed us around the ward through live broadcast*, *contacted patients in advance*, *guided us in doing online consultations*, *etc*. *All of these gave us the feeling of being there”* (Participant 15).

*“The teachers made real clinical videos for us*, *such as videos of nurses’ bedside shift*. *When I watched a video*, *I would imagine myself as the person in the video”* (Participant 13).

### Main theme 4: Limitations of e-internships

Despite the benefits above, limitations of e-internships are also obvious in these students’ eyes.

#### Sub-theme 1: Limitations of practice opportunities

The reason why the internship is a privileged moment for nursing students is that it provides students the opportunity to integrate academic knowledge into professional clinical practice. Undoubtedly, the lack of clinical practice is the biggest limitation of e-internships.

*“There was no chance for us to practice*. *This is the biggest shortcoming of e-internships*. *I truly hope to perform some simple exercises in a real clinical setting”* (Participant 4).

During on-site internships, one can encounter patients with different types of diseases, different personalities, and different cultural backgrounds. The complex clinical environment will push students to respond accordingly. However, e-internships cannot provide these learning opportunities.

“*During e-internships*, *I have no chance to reach different kinds of patients*, *different complex clinical environments”* (Participant 13).

#### Sub-theme 2: Deficits in e-teaching ability

Although knowledgeable about the didactics of traditional internships, not all of clinical nursing teachers were well prepared for changes brought about by the epidemic. From the descriptions of the students, we knew that the e-internship exposed some deficiencies in the teachers’ e-teaching ability.

*“Some teachers lack experience in using online teaching software*, *such as Tencent Meeting and Dingtalk*, *resulting in waste of time and low teaching efficiency”* (Participant 6).

Some students pointed out that some teachers lack online interaction.

*“Some teachers spoke too fast and lacked interaction with students*, *and the classroom atmosphere was dreary*” (Participant 1).

Some participants felt that the teaching schedule was a bit unreasonable.

“*In my opinion*, *the difficulty of the course and the teaching speed should be gradual*.*"* (Participant 7).

Furthermore, many participants suggested that more case analysis classes were needed.

*“I hope they can present more clinical nursing case analysis*, *let us students analyze independently first*, *and then give us instructions” (*Participant 15).

*“I suggest that teachers use clinical cases to explain the main points of nursing; doing so would not only increase the interest of us students but also impress us more deeply”* (Participant 7).

Some students expressed their concerns about classroom discipline and advised teachers to take targeted measures.

*“Without the supervision of teachers*, *students with low self-discipline would neglect their learning*, *thereby leading to poor learning effects*.*”* (Participant 3).

*“More tasks could be assigned after class to test whether students master what they were being taught*.*”* (Participant 7).

#### Sub-theme 3: Deficits in e-teaching resources

To cope with the consequences of COVID-19, each department quickly developed its e-internship resource library according to the internship syllabus. However, due to experience and time constraints, e-internship resources were still scarce. Students were eager to obtain more new knowledge and skills about their future jobs.

“*We hope that teachers can introduce more new technologies and new developments in specialties*” (Participant 4).

*“The online classes should add more videos about specialized diseases*, *especially videos of nursing practices*.” (Participant 17).

*“If possible*, *I suggest they demonstrate the real situation to us through live broadcast or take real videos to help us understand the responsibilities of each post*. *We are truly very curious about a nurse’s daily work”* (Participant 13).

#### Sub-theme 4: Limitations of the learning platforms

Currently, e-learning in China is held on two main platforms, namely, Tencent Meeting and Dingtalk. However, students pointed out that the network was unstable during online classes, thus affecting learning effectiveness.

“*Unstable network*, *loud noise*, *intermittent picture and sound affected the learning experience”* (Participant 15).

*“I truly suggest that teachers utilize a better learning platform*, *for example*, *Dingtalk*. *This platform has a replay function*, *allowing students to review”* (Participant 2).

## Discussion

This study explored the perceptions and experiences of undergraduate nursing students who completed an eight-week e-internship program during the COVID-19 pandemic by using phenomenological methods. Four themes were captured: perceived images of clinical nurses in e-internships, psychological experience, perceived benefits of e-internships, and perceived limitations of e-internships.

The findings of this study showed that during their e-internships, the students gained more knowledge about the images of nurses. In their opinion, nurses are “dedicated and hardworking” and “professional and skillful”. The students appreciated their teachers’ dedication and praised their teachers’ clinical skills and teaching abilities. Robert and his colleagues [20] discovered similar results in their qualitative research, all participants praised the professionalism, courage and humanity of teachers during this crisis. Images of clinical nursing teachers are directly related to what kind of nurses the students will become in the future [21]. In this study, several participants said that they admire their teachers and expected to eventually become nurses like them. Practically, for nursing students, internships are a process of learning and a process of exploring their own future work [22]. The students’ image of nurses is probably what the students perceive of themselves in their future career. With a positive image in their mind, the students will attach a strong sense of professional identity to the job they are committed to and thereby better motivate their self-efficacy to achieve this image. Otherwise, they are likely to show little interest in becoming nurses [23, 24]. Therefore, it is very important to culture and select nursing teachers with outstanding qualities and abilities for nursing students, whether the students receive in-person or e-internship instruction.

When students are faced with the sudden suspension of the internship, it is normal for them to feel regretful and anxious. In our study, few students worried about being infected. In contrast, they were anxious to carry out on-site clinical internships and even expected to play a role in preventing and controlling the epidemic. This finding differs from that of Cristina’s study in Portugal [25]; The aforementioned study showed that during their clinical internships, nursing students were afraid of becoming infected, coming out of lockdown and being in contact with people and providing care. This difference may result from the different epidemic times and degrees in different countries. In July and August 2020, COVID-19 was basically controlled in China [26], while in other countries, the epidemic is still developing rapidly [27].

The feeling of uncertainty is common in the participants’ descriptions of their psychological experiences during the e-internships [28–30]. The participants’ sense of uncertainty comes from two main sources. One is the limitations of e-internships, and the other is the epidemic situation. Obviously, the practical ability of nurses needs to be tested in clinical practice, but in e-internships, nursing students are deprived of this opportunity. In e-internships, nursing students also have no chance to communicate with teachers and patients face to face or to receive direct guidance and supervision from teachers, thus increasing the sense of uncertainty about the effect of e-internships [29, 30]. In addition, the sudden occurrence of the epidemic made nursing students realize their insignificance and their helplessness in the current situation and produced in them a sense of uncertainty [31, 32]. To reduce the sense of uncertainty, teachers should strengthen communication with students, create conditions for them to communicate with patients, convey more positive developments about the epidemic to them and conduct psychological interventions when necessary [28]. Certainly, the most useful method is to resume their most anticipated clinical on-site internships as soon as possible if conditions permit, to purposefully strengthen the assessment of what they have learned during e-internships, and to conduct targeted actions when necessary.

Due to the sudden outbreak of the epidemic and the high mortality rate, it is indeed too risky to carry out on-site internships before the epidemic is controlled. Although there is no way to fully replace the experience of on-site internships, students in this study believed that e-internships were the most reasonable arrangements the hospital could make for them in such a special and critical situation. In our study, the content of the e-internship is student-centered and offers a great deal of flexibility in terms of location. In addition, teachers used many online tools, such as a combination of audio, videos, text, PPT, live broadcast, and so on, to reach out to their students. Moreover, students can watch a video repeatedly to gain a deeper understanding of what teachers said. All these factors made e-internships possible and effective for nursing students. Thus, although these students regretted not being able to participate in traditional internships, the students still showed good acceptance of e-internships and perceived that they achieved much during e-internships.

We are not the only ones to support e-internships. At the height of the epidemic, almost the whole world has suspended face-to-face learning and on-site internships. e-internships, virtual internships [33, 34], and distant teaching [35, 36], similar learning methods based on information technology, have been employed unprecedentedly in the world. Michael et al. [36] found that web-based surgical skills learning is a feasible alternative for face-to-face surgical skills teaching. Hao [37] concluded that standalone digital education modalities were as effective as conventional learning for knowledge and practice. E-internships have also been applied to dental education [38] and urological education [32] and have achieved positive results. Based on these findings, we have reasons to believe that e-internships are promising and worthy of promotion. However, as stated by the participants in this study, there are still many limitations that need to be addressed to make the e-internships better.

In this study, participants presented four main limitations about their e-internships, namely, limitations of practice opportunities, deficits in e-teaching ability, deficits in e-teaching resources and limitations of the learning platforms. The major reason there were so many limitations may be that there is a lack of standards for quality, quality control, development of e-resources, and e-content delivery [39]. Therefore, hospitals should first formulate guidelines and standards for e-internships according to the characteristics of nursing students. Departments and teachers will know how to use these guidelines and standards to improve themselves. The e-teaching ability of teachers needs to be emphasized [39, 40]. The most basic requirement for teachers is to be proficient in operating the network teaching platform. In addition, teachers should present the curriculum in various formats, such as video, audio, and text, to increase students’ interests [11]. Students want two-way interaction; therefore, teachers should fully use the video function of the online platform to interact with students, such as by “calling the roll” and "asking questions”, to improve students’ sense of participation. It would be better if teachers complemented their lectures with video chats, virtual meetings, and so on, to obtain immediate feedback and maintain a personal connection with the students.

For the learning platform, with the continuous update of technology, Tencent Meeting and Dingtalk have become functionally stable learning platforms in China. Of course, it would be better if the institution or hospital could establish its own learning platform. With its own learning platform, each department can build its own resource library, and teachers, students and other relevant personnel can log in to their accounts for free, thus bringing e-learning into a more developed era.

The limitations of our study must be acknowledged. First, only nursing students were interviewed, related nursing teachers and nursing managers were excluded, which limited the reference significance of this research. In addition, e-internships were conducted from July to August, but our interviews started in October. There may be some time deviations in students’ perceptions and experiences.

## Conclusion

With the rapid developments of technology, e-learning has been a trend. Carrying out e-internships is a way not only to deal with crisis situations but also to explore more possibilities for internships. In this study, our findings suggest that the e-internship is a suitable method for training and cultivating undergraduate nursing students’ clinical knowledge and skills during a crisis. To enhance the efficiency of e-internships, guidelines and standards should be formulated, and effective measures should be taken to build better e-internship platforms. In the future, we can try to combine on-site internships with e-internships, thereby fully using their advantages, and to improve the efficiency of internships as much as possible.

## References

[1] Gao Y, Wang C, An W, et al. The actuality and enlightenment of nursing student practice education in China and America, Chin J Nurs Educ, 2018;15(8): 628–631.

[2] Zou Y, Chen J. Development and Enlightenment of Nursing student Education in International First-class Universities, Chin J Nurs Educ, 2019;16 (6):469–472.

[3] Ayaz-Alkaya S, Yaman-Sözbir S, Bayrak-Kahraman B. The effect of nursing internship program on burnout and professional commitment. Nurse education today, 2018, 68: 19–22. doi: 10.1016/j.nedt.2018.05.020 29870870

[4] You L, Ke Y, Zheng J, et al. The development and issues of nursing education in China: A national data analysis. Nurse Education Today 2015; 35(10):310–314. doi: 10.1016/j.nedt.2014.10.004 25456256

[5] Huang L, Lei W, Xu F, et al. Emotional responses and coping strategies in nurses and nursing students during Covid-19 outbreak: A comparative study. PloS one, 2020, 15(8): e0237303. doi: 10.1371/journal.pone.0237303 32764825 PMC7413410

[6] Elhaty I A, Elhadary T, Elgamil R, et al. Teaching university practical courses online during COVID-19 crisis: A challenge for elearning. J. Crit. Rev, 2020, 7(8): 1–10.

[7] Thapa P, Bhandari S L, Pathak S. Nursing students’ attitude on the practice of e-learning: A cross-sectional survey amid COVID-19 in Nepal. PLoS ONE, 2021, 16(6). doi: 10.1371/journal.pone.0253651 34166444 PMC8224981

[8] Radha R, Mahalakshmi K, Kumar V, et al. E-Learning during lockdown of Covid-19 pandemic: A global perspective. International journal of control and automation, 2020, 13(4): 1088–1099.

[9] Diab G, Elgahsh N F. E-learning during COVID-19 pandemic: Obstacles faced nursing students and its effect on their attitudes while applying it. American Journal of Nursing, 2020, 9(4): 300–314.

[10] Alqahtani N, Innab A, Bahari G. Virtual Education During COVID-19: Exploring Factors Associated With E-Learning Satisfaction Among Saudi Nursing Students. Nurse educator, 2021, 46(2): E18–E22. doi: 10.1097/NNE.0000000000000954 33234836

[11] Singh H K, Joshi A, Malepati R N, et al. A survey of E-learning methods in nursing and medical education during COVID-19 pandemic in India. Nurse education today, 2021, 99: 104796. doi: 10.1016/j.nedt.2021.104796 33607513 PMC7865095

[12] Guven Ozdemir N, Sonmez M. The relationship between nursing students’ technology addiction levels and attitudes toward e‐learning during the COVID‐19 pandemic: A cross‐sectional study. Perspectives in Psychiatric Care, 2020. doi: 10.1111/ppc.12710 33305866

[13] Lopez K A, Willis D G. Descriptive versus interpretive phenomenology: Their contributions to nursing knowledge. Qualitative health research, 2004, 14(5): 726–735. doi: 10.1177/1049732304263638 15107174

[14] Todres L. Clarifying the life-world: Descriptive phenomenology. Qualitative research in health care, 2005: 104–124.

[15] Braun V, Clarke V. To saturate or not to saturate? Questioning data saturation as a useful concept for thematic analysis and sample-size rationales. Qualitative research in sport, exercise and health, 2021, 13(2): 201–216.

[16] Namey E, Guest G, McKenna K, et al. Evaluating bang for the buck: a cost-effectiveness comparison between individual interviews and focus groups based on thematic saturation levels. American Journal of Evaluation, 2016, 37(3): 425–440.

[17] Colaizzi PF. Psychological research as the phenomenologist views it. In: Valle RS, King M, eds. Existential- phenomenological alternatives for psychology. New York, NY: Oxford University Press, 1978: 48–71.

[18] Tong A, Sainsbury P, Craig J. Consolidated criteria for reporting qualitative research (COREQ): a 32-item checklist for interviews and focus groups. International journal for quality in health care, 2007, 19(6): 349–357. doi: 10.1093/intqhc/mzm042 17872937

[19] Lincoln Y S, Guba E G. Naturalistic inquiry Newbury Park. Cal.: Sage, 1985:289–331.

[20] Lovrić R, Farčić N, Mikšić Š, et al. Studying during the COVID-19 pandemic: A qualitative inductive content analysis of nursing students’ perceptions and experiences. Education Sciences, 2020, 10(7): 188.

[21] Garbrah W, Kankkunen P, Välimäki T. Gerontological nurse teachers’ abilities and influence on students’ willingness in older people nursing: A cross-sectional, correlational survey. Nurse education today, 2020, 90: 104461. doi: 10.1016/j.nedt.2020.104461 32408244

[22] Marañón A A, Pera M P I. Theory and practice in the construction of professional identity in nursing students: a qualitative study. Nurse education today, 2015, 35(7): 859–863. doi: 10.1016/j.nedt.2015.03.014 25863650

[23] Monforte-Royo C, Fuster P. Coronials: Nurses who graduated during the COVID-19 pandemic. Will they be better nurses?. Nurse education today, 2020, 94: 104536. doi: 10.1016/j.nedt.2020.104536 32801065 PMC7387935

[24] Shengxiao N I E, Chao S U N, Lei W, et al. The Professional Identity of Nursing Students and Their Intention to Leave the Nursing Profession During the Coronavirus Disease (COVID-19) Pandemic. Journal of Nursing Research, 2021, 29(2): e139. doi: 10.1097/jnr.0000000000000424 33534354

[25] Baixinho C L, Ferreira Ó R. Being a nursing student in times of COVID-19. Escola Anna Nery, 2021, 25.

[26] The latest situation of the novel coronavirus pneumonia epidemic: As of 24:00 on August 30, http://www.nhc.gov.cn/xcs/yqtb/202008/1eb982b3f8024d9e9f5ed461351385bb.shtml

[27] World Health Organization https://worldhealthorg.shinyapps.io/covid/

[28] Valdez G F D. Dilemma, Uncertainties and Fear: Nursing Faculty & Students Clinical Exposure and Training in a COVID-19 Situations. Saudi J Nurs Health Care, 2021, 4(3): 75–76.

[29] Ramos-Morcillo A J, Leal-Costa C, Moral-García J E, et al. Experiences of nursing students during the abrupt change from face-to-face to e-learning education during the first month of confinement due to COVID-19 in Spain. International journal of environmental research and public health, 2020, 17(15): 5519. doi: 10.3390/ijerph17155519 32751660 PMC7432480

[30] Cengiz Z, Gurdap Z, Işik K. Challenges experienced by nursing students during the COVID‐19 pandemic. Perspectives in Psychiatric Care, 2021. doi: 10.1111/ppc.12923 34350616 PMC8447074

[31] Elsharkawy N B, Abdelaziz E M. Levels of fear and uncertainty regarding the spread of coronavirus disease (COVID-19) among university students. Perspect. psychiatr. care, 2021, 57(3):1356–1364. doi: 10.1111/ppc.12698 33258117 PMC7753423

[32] Chen W, Chen J, Hu J, et al. The Professional Activities of Nurse Managers in Chinese Hospitals: A Cross-Sectional Survey in Hunan Province. Journal of Nursing Management, 2021, 29,145–151. doi: 10.1111/jonm.13110 32715553

[33] Mikhail D, Margolin E J, Sfakianos J, et al. Changing the Status Quo: Developing a Virtual Sub-Internship in the Era of COVID-19. Journal of surgical education, 2021. doi: 10.1016/j.jsurg.2021.03.007 33896734 PMC8419923

[34] Monday L M, Gaynier A, Berschback M, et al. Outcomes of an online virtual boot camp to prepare fourth-year medical students for a successful transition to internship. Cureus, 2020, 12(6). doi: 10.7759/cureus.8558 32670695 PMC7357344

[35] He M, Tang X, Zhang H, et al. Remote clinical training practice in the neurology internship during the COVID-19 pandemic. Medical education online, 2021, 26(1): 1899642. doi: 10.1080/10872981.2021.1899642 33685381 PMC7946031

[36] Co M, Chu K M. Distant surgical teaching during COVID‐19‐A pilot study on final year medical students. Surgical practice, 2020, 24(3): 105–109. doi: 10.1111/1744-1633.12436 32837531 PMC7361811

[37] Hao X, Peng X, Ding X, et al. Application of digital education in undergraduate nursing and medical interns during the COVID-19 pandemic: A systematic review. Nurse education today, 2021: 105183. doi: 10.1016/j.nedt.2021.105183 34741918 PMC8545701

[38] Chang T Y, Hong G, Paganelli C, et al. Innovation of dental education during COVID-19 pandemic. Journal of Dental Sciences, 2021, 16(1): 15–20. doi: 10.1016/j.jds.2020.07.011 32839668 PMC7437532

[39] Dhawan S. Online learning: A panacea in the time of COVID-19 crisis. Journal of Educational Technology Systems, 2020, 49(1): 5–22.

[40] Hu S, Chen J, Jiang R, et al. Caring ability of nursing students pre- and post-internship: A longitudinal study. BMC Nursing, 2022, 21(133): 1–7. doi: 10.1186/s12912-022-00921-2 35644615 PMC9150307

